# Bias due to censoring of deaths when calculating extra length of stay for patients acquiring a hospital infection

**DOI:** 10.1186/s12874-018-0500-3

**Published:** 2018-05-30

**Authors:** Shahina Rahman, Maja von Cube, Martin Schumacher, Martin Wolkewitz

**Affiliations:** 1 Institute of Medical Biometry and Statistics, Faculty of Medicine and Medical Center - University of Freiburg, Freiburg, Germany; 2 Freiburg Center of Data Analysis and Modelling, University of Freiburg, Eckerstr. 1, Freiburg, 79104 Germany; 3 Department of Statistics, Texas A&M University, 3143 TAMU, 77843-3143, College Station, Texas, USA

**Keywords:** Bias, Censored deaths, Extra length of stay, Hospital acquired infection, Multistate model

## Abstract

**Background:**

In many studies the information of patients who are dying in the hospital is censored when examining the change in length of hospital stay (cLOS) due to hospital-acquired infections (HIs). While appropriate estimators of cLOS are available in literature, the existence of the bias due to censoring of deaths was neither mentioned nor discussed by the according authors.

**Methods:**

Using multi-state models, we systematically evaluate the bias when estimating cLOS in such a way. We first evaluate the bias in a mathematically closed form assuming a setting with constant hazards. To estimate the cLOS due to HIs non-parametrically, we relax the assumption of constant hazards and consider a time-inhomogeneous Markov model.

**Results:**

In our analytical evaluation we are able to discuss challenging effects of the bias on cLOS. These are in regard to direct and indirect differential mortality. Moreover, we can make statements about the magnitude and direction of the bias. For real-world relevance, we illustrate the bias on a publicly available prospective cohort study on hospital-acquired pneumonia in intensive-care.

**Conclusion:**

Based on our findings, we can conclude that censoring the death cases in the hospital and considering only patients discharged alive should be avoided when estimating cLOS. Moreover, we found that the closed mathematical form can be used to describe the bias for settings with constant hazards.

**Electronic supplementary material:**

The online version of this article (10.1186/s12874-018-0500-3) contains supplementary material, which is available to authorized users.

## Background

Change in length of stay (cLOS) in hospital is a key outcome when studying the health impact and economic consequences of hospital acquired infections (HIs). A patient with an HI is likely to stay longer in the hospital, incurring extra costs. Thus, appropriately quantifying the cLOS in hospitals (in days) due to HIs is crucial for economical and policy decision making. However, a correct estimation of cLOS is challenging and prone to bias. This is not only because HIs are time-dependent covariates but also because there are two possible controversy outcomes, namely in-hospital death and discharge from the hospital alive.

Barnett et al. [1] used a multi-state model to show the occurrence of substantial bias in estimating cLOS when studies fail to treat HIs as a time-dependent exposure (this bias is known as ’time-dependent bias’).

Brock et al. [2] found that the way in which mortality is handled while investigating other time-related outcomes (such as discharge alive) influences the estimate of cLOS. They contrasted two ad-hoc approaches. In the first approach they restricted the analysis to the patients who survived. In the second approach, individuals who died were right-censored at the longest possible follow-up time. They concluded that the two methods can potentially give different results for the same data. Brock et al. argue that this could lead to conflicting conclusions, unless the investigators are aware of the differences between the estimators.

In many studies patients who are dying in the hospital are censored at the time of death to study the cLOS in hospitals due to HIs. One recent example is a study by Noll et al. [3]. They calculated cLOS by censoring the outcome of patients who died in the hospital, had ventilator dependent respiratory failure, or withdrew from the study. Another recent study by Guerra et al. [4] censored the patients who were not discharged from the hospital to their usual residence within the study period, namely death cases or patients that were transferred, to investigate the cLOS due to HIs. Zuniga et al. [5] censored death cases and analysed the cLOS considering information only of the patients who were discharged alive.

However, death in the hospital is informative censoring and should be treated in a competing risks framework as proposed by Schulgen et al. [6]. In this article, we show that treating death-cases in the hospital as non-informative censoring can lead to biased estimates of cLOS.

It may be argued that the mortality rates in hospitals are usually not very high, as most of the patients are discharged alive. Thus, using only the information of the patients discharged alive might lead to reasonable estimation of cLOS in many cases. However, the efficiency of such an estimator might be questionable. Moreover, in intensive-care units (ICUs) where HIs are a serious problem, the mortality can reach up to 30-50%. This is for instance the case for ventilated and critically-ill patients. Since cLOS is often used to calculate costs as costs are driven by bed days, we argue that the costs of a hospital stay are not affected by the status of the patient at the end of stay.

A reason for censoring the death cases may be the wish to give cLOS (and cost) estimates for a hospital-population which is discharged alive. Therefore, we propose to follow the approach by Allignol et al. [7]. They suggest to first use the combined endpoint ’discharge (dead or alive)’ to calculate the overall cLOS (which can also be used for a cost analysis) and second, to distinguish the impact of HIs on cLOS between patients discharged alive and patients deceased. Based on this approach, studies censoring the patients at the time of their death are prone to bias.

To understand and quantify the difference of the competing risks and the censoring approach, we assume the simplified setting of constant hazards. For this setting, we derive an analytical expression for the difference of cLOS estimated as proposed by Allignol et al. [7] and cLOS estimated by censoring the deceased patients. This analytical expression can be used to investigate and analyse the magnitude of the bias that occurs when estimating cLOS by censoring patients that die. Motivated by the work of Joly et al. [8] and Binder and Schumacher [9], we systematically investigate the bias with respect to “differential mortality”. In their setting differential mortality is a term which defines the difference in the rate of mortality of the patients with and without the infection. They consider an illness-death model where HI is an intermediate event between admission and death. In our setting we have two competing outcomes (death in hospital or discharge alive). HI is an intermediate event between admission and death or discharge which ever comes first. Therefore, we have considered two kinds of “differential mortality” in the time-constant hazards set up, which affects the absolute mortality risk of a patient: 1. “direct differential mortality”, when the death hazards with and without the infection differ while the discharge hazards with and without the infection remain the same. 2. “indirect differential mortality”, when discharge hazard rates with and without the infection differ while death hazards with and without the infection remain the same. The type of differential mortality can be studied with cause-specific Cox proportional hazards models for death and discharge with HI as time-dependent covariate.

Moreover, we compare the estimate of cLOS from the biased model with the cLOS attributed to patients discharged alive. To do so, we use the formula derived by Allignol et al. [10]. They propose a simple method to split the extra days due to HIs in the hospital into days attributable to patients that die and attributable to those that are discharged alive. This can be done for both homogeneous Markov models and for time-inhomogeneous Markov models. The methods for the time-inhomogeneous model are implemented in the R-packge *etm*, developed by Allignol et al. [7].

In “Methods” section part 1 we shortly discuss the formulas to estimate cLOS with the two approaches under the constant hazards assumption. In “Methods” section part 2, we aim to provide a proper analytical expression of the potential bias in estimating the cLOS due to HIs when the information on the death cases in the hospital is censored. Assuming a time-homogeneous Markov model, where the transition hazards are time-independent, we systematically explore the amount and direction of the bias. In “Results and discussion” section, we illustrate the real-world relevance of the bias by analysing a random subset of the SIR-3 prospective cohort study on hospital acquired pneumonia in ICUs in Berlin, Germany. For the real data analysis, we estimate the cLOS by applying the method for time-inhomogenuous Markov models developed by Allignol et al. [7], which is based on the Aalen-Johansen estimator. The paper ends with a short discussion in “Conclusion” section.

## Methods

### Multi-state model for hospital infections

We focus on estimating the cLOS in the hospital due to HIs. We study the amount of bias which can occur when estimating the cLOS by treating patients that die as censored.

**Fig. 1:**
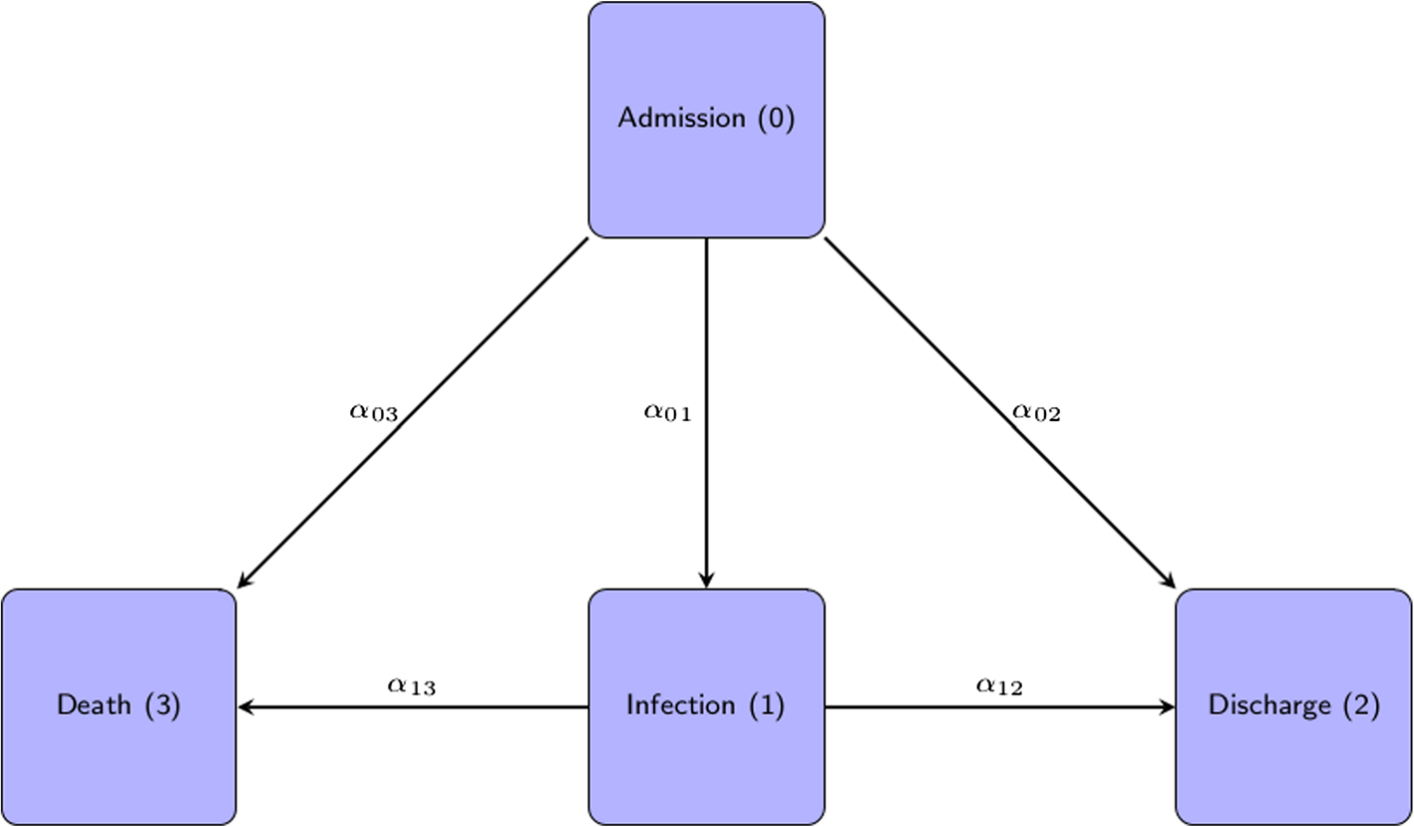
Model A: The four state Multistate Model; 0 is “Admission” without hospital acquired infection (HI); 1 is hospital acquired “Infection”; 2 is the status of the patients who are “Discharged Alive” and 3 is the “Death” of the patient in the hospital. The constant hazard rates, *α*_01_ is the hazard rate to acquire the hospital infection during the hospital stay; *α*_02_ is the hazard rate to be discharged alive without the HI; *α*_03_ is the hazard rate to dead without the HI and *α*_12_ is the hazard rate to be discharged alive after the HI; *α*_13_ is the hazard rate to be dead after the HI

To do so, we describe the data setting with a multi-state model as proposed by e.g. [7]. Figure 1 displays this model (model A), which is a multi-state model with states, 0= admission, 1= infection, 2= discharge alive and 3= death. For simplicity we assume that the hazard rates are constant over time so that we can focus on the key points concerning the censoring of the death cases. We denote *α*_*ij*_(*t*)=*α*_*ij*_ as the hazard of moving from state “i” to state “j”. An example hazard is,

$${} \alpha_{01}\!(t)\cdot\Delta t \!\approx\! P(\text{HI acquired by time t} \,+\, \Delta t | \text{no HI up to time t}). $$

The actual hazard *α*_01_(*t*) is obtained by taking limits as *Δt*→0. We define the hazard rates, *α*_01_= infection hazard rate; *α*_02_= discharge hazard rate without infection; *α*_03_= death hazard rate without infection; *α*_12_= discharge hazard rate with infection and *α*_13_= death hazard rate with infection. Under a constant hazards assumption, one estimates *α*_*ij*_ by using the maximum likelihood estimator

1 $$ \hat{\alpha}_{ij} = \frac{\text{number of i} \to \text{j transitions}}{\text{person-time in state i}}.   $$

Under this model the mean sojourn time of an infected patient in the hospital is $\frac {1}{\alpha _{12}+\alpha _{13}}$ and of an uninfected patient it is $\frac {1}{\alpha _{01}+\alpha _{02}+\alpha _{03}}$. We write *X*_*t*_ for the state occupied by the patient at time t. At a time point *t*, the patient status *X*_*t*_∈{0,1,2,3}. By definition, all individuals start in the initial state 0 of being alive in the hospital and free of HI, i.e., *X*_0_=0. We denote *T* as the smallest time at which the process is in an absorbing state, *T*=inf{*t*:*X*_*t*_∈{2,3}}. Eventually, end of the hospital stay occurs when *X*_*T*_∈{2,3}.

To evaluate the impact of HIs on the subsequent hospital stay, Schulgen and Schumacher (1996) [6] suggested to consider the difference of the expected subsequent stay given infectious status at time s, *ϕ*(*s*)=*E*(*T*|*X*_*s*_=1)−*E*(*T*|*X*_*s*_=0). Schulgen and Schumacher called *ϕ*(*s*) the ’expected extra hospitalization time of an infected individual dependent on time s’. In our setting, the process follows a homogeneous Markov model. Allignol et al. [7] studied the cLOS for model A (Fig. 1) mathematically and found that cLOS does not depend on the time s in the homogeneous case. The cLOS can therefore be expressed as

2 $$ {} \text{CLOS}_{true} = \phi(s) = \left[\frac{\alpha_{02} + \alpha_{03}}{\alpha_{12} + \alpha_{13}}-1\right]\times \frac{1}{ \alpha_{01} + \alpha_{02} + \alpha_{03}}  $$

Furthermore, Allignol et al. provided a formula to separate the estimation of the cLOS for the discharged patients and the deceased patients under the constant hazard set up. This formula is given by

3 $$ {\begin{aligned} {} \text{CLOS} &= \text{CLOS(due to discharged alive)} \\ &\quad+ \text{CLOS(due to deaths)}\\ &= \frac{\alpha_{12}}{\alpha_{12} + \alpha_{13}}\times \text{CLOS} + \frac{\alpha_{13}}{\alpha_{12} + \alpha_{13}}\times \text{CLOS} \end{aligned}}  $$

Hence, we can separately estimate cLOS attributable to patients discharged alive and cLOS attributable to death cases by plugging in the estimates of the constant hazards obtained with (1).

Model B results from model A when treating death cases as censored. In contrast to model A, patients that die are assumed to remain under the same risk of being discharged alive as patients that are still in the hospital. While the discharge hazards of model A and B are the same, the absolute chance of discharge alive in model A depends on the competing risk death and therefore differs from the discharge probability modelled in model B. To derive the cLOS that results from model B, we apply the formula proposed by Allignol et al. which is then

4 $$ \text{CLOS}^{*} = \left[\frac{\alpha_{02}}{\alpha_{12}}-1\right]\frac{1}{\alpha_{01} + \alpha_{02}}.  $$

### Analytic expression for the bias

Our focus is on investigating the bias in cLOS when the information of the patients that die is censored. Using the formulas in Eqs. (2) and (4), we deduce that the bias in cLOS due to censoring is,

5 $$ {\begin{aligned} \text{CLOS}^{*} - \text{CLOS}_{true} =& \frac{\alpha_{03}(\alpha_{02} - \alpha_{12})}{\alpha_{12}(\alpha_{01} + \alpha_{02} + \alpha_{03})(\alpha_{01} + \alpha_{02})} \\ &+\frac{(\alpha_{02}\alpha_{13}- \alpha_{03}\alpha_{12})}{\alpha_{12}(\alpha_{01} + \alpha_{02} + \alpha_{03})(\alpha_{12}+\alpha_{13})}\\ =&\frac{\alpha_{03}(\alpha_{02} \,-\, \alpha_{12})}{\alpha_{12}\alpha_{0\cdot}\alpha^{*}_{0\cdot}} \,+\, \frac{(\alpha_{02}\alpha_{13}\,-\, \alpha_{03}\alpha_{12})}{\alpha_{0\cdot}\alpha_{1\cdot}\alpha_{12}}, \end{aligned}}  $$

where *α*_0·_=*α*_01_+*α*_02_+*α*_03_, $\alpha ^{*}_{0\cdot } = \alpha _{01} + \alpha _{02}$, *α*_1·_=*α*_12_+*α*_13_ and $\alpha ^{*}_{1\cdot } = \alpha _{12}$. The formula shows that the bias depends on the product of the mean LOS in state 0 (*α*_0._) and a term depending on all hazards. The second term determines the direction of the bias which could be positive or negative. In the following, we study the bias in specific settings which we call differential mortality. We define “direct differential mortality” as the setting where the discharge hazards *α*_02_ and *α*_12_ are the same but the death hazards *α*_03_ and *α*_13_ differ. In contrast, “indirect differential mortality” is described by equal death hazards but different discharge hazards. Of note, due to the competing risk situation both settings influence - directly or indirectly - the overall hospital mortality. We define *Δ*_1_=*α*_13_−*α*_03_ and *Δ*_2_=*α*_02_−*α*_12_ and emphasize that both quantities are likely to be positive because infected patients often have a higher mortality hazard and a lower discharge hazard, i.e., they stay longer in the hospital.

A formal mathematical derivation of the bias can be found in Additional file 1.

#### No differential mortality

The bias predominately depends on the hazard rates. In the following we study the magnitude of the bias under differential mortality. When there is no differential mortality, that is, no difference between the death hazards with and without infection and no difference between the discharge hazards with and without infection, *Δ*_1_=*α*_13_−*α*_03_=0 and *Δ*_2_=*α*_02_−*α*_12_=0, the bias becomes 0. The following formula can be used to obtain an idea of the magnitude and the direction of the bias for given values of the hazard functions when the death cases are censored.

#### Direct differential mortality

Under direct differential mortality, there is a non-zero difference between the death hazards with and without infection while the discharge hazards with and without infection are the same, that is *Δ*_2_=*α*_02_−*α*_12_=0 and *Δ*_1_=*α*_13_−*α*_03_≠0. Then, the bias can be expressed as

6 $$\begin{array}{*{20}l} \text{CLOS}^{*} - \text{CLOS}_{true} &= (\alpha_{13} - \alpha_{03})\cdot\frac{1}{\alpha_{0\cdot}}\cdot\frac{1}{\alpha_{1\cdot}} \\ &= \Delta_{1}\cdot\frac{1}{\alpha_{0\cdot}}\cdot\frac{1}{\alpha_{1\cdot}}.  \end{array} $$

The bias changes with *Δ*_1_. Moreover, as $\frac {1}{\alpha _{0\cdot }}$ and $\frac {1}{\alpha _{1\cdot }}$ are the average sojourn time in state 0 and state 1 of uninfected and respectively infected patients, the bias also increases when the average sojourn times increase.

#### Indirect differential mortality

Under indirect differential mortality, there is a non-zero difference between the discharge hazards with and without infection while the death intensities with and without infection are the same, that is *Δ*_1_=*α*_13_−*α*_03_=0 and *Δ*_2_=*α*_02_−*α*_12_≠0. Then, the bias is

7 $$\begin{array}{*{20}l} {} \text{CLOS}^{*}\! -\! \text{CLOS}_{true} &\,=\, \left(\alpha_{02} - \alpha_{12}\right) \!\cdot\!\frac{1}\!\cdot\!\frac{1}{\alpha_{1\cdot}}\!\cdot\!\frac{\alpha_{03}\left(\alpha_{0\cdot} + \alpha_{12}\right)}{\alpha_{12}\alpha^{*}_{0\cdot}} \\ &= \Delta_{2} \cdot\frac{1}{\alpha_{0\cdot}}\cdot\frac{1}{\alpha_{1\cdot}}\cdot\frac{\alpha_{03}\left(\alpha_{0\cdot} + \alpha_{12}\right)}{\alpha_{12}\alpha^{*}_{0\cdot}}. \end{array} $$

The bias changes with *Δ*_2_. The bias also increases with the average waiting time in state 0 and in state 1. Again, in most of the real world situations, we observe *Δ*_2_>0, which means the infected patients have lower discharge rates than the uninfected ones. Then, the bias is positive which leads to an overestimation of the cLOS.

The derived analytical expressions demonstrate for a simplified setting (constant hazards, differential mortality) how estimation of cLOS is influenced when information of the death cases is censored. Only in the situation where HIs have neither an effect on the death hazards nor on the discharge hazards, the bias is avoided. Otherwise, the bias increases with increasing magnitude of the differential mortality.

## Results and discussion

**Fig. 2:**
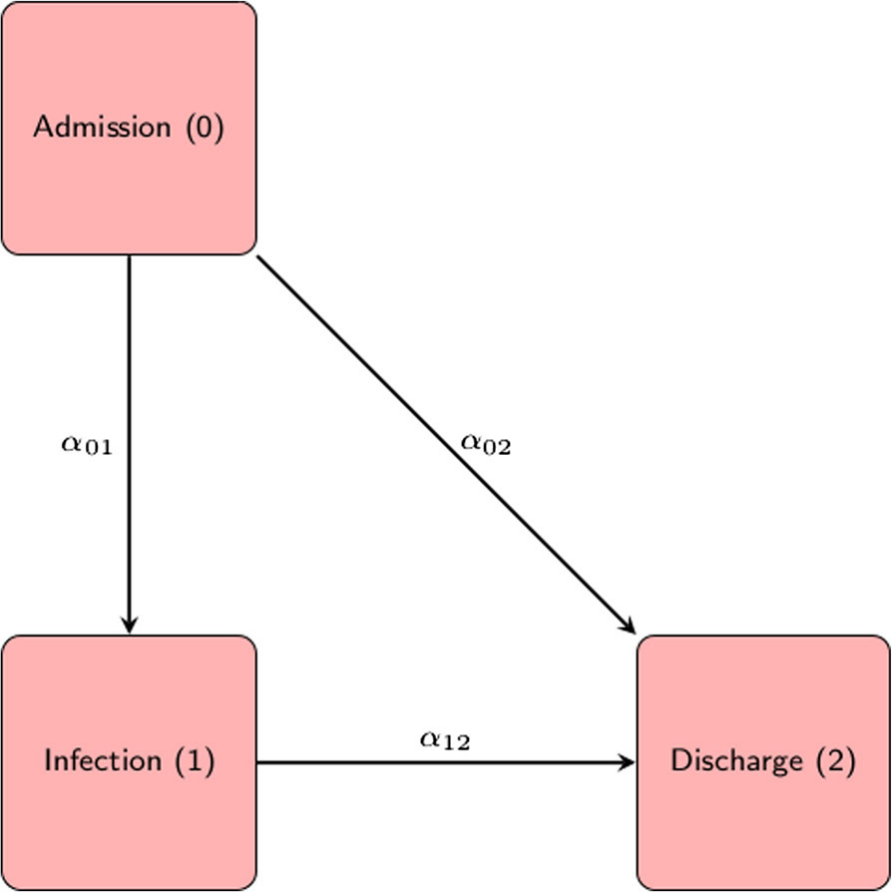
Model B: Multistate Model resulting from censoring the death cases; 0 is the “Admission” state; 1 is HI; 2 is the status of the patients who are “Discharged Alive” and information on rest of the patients are “Censored”. The constant hazard rates that can be calculated from the model, *α*_01_ is the hazard rate to acquire a HI infection during the hospital stay; *α*_02_ is the hazard rate to be discharged alive without the HI; and *α*_12_ is the hazard rate to be discharged alive after the HI

To show the real world relevance of our findings, we apply the method to a data example. The constant hazards assumption is a facilitating way to compare the estimands of cLOS resulting from model A and model B. However, for real data application it is often too restrictive. Therefore, in our data example we compare models A (Fig. 1) and B (Fig. 2) both under the constant hazards assumption (time-homogeneous Markov model) and more generally under a time-inhomogeneous Markov model.

We consider a subset of the SIR-3 cohort study from the Charite university hospital in Berlin, Germany, with prospective assessment of data to examine the effect of HIs in intensive care (Beyersmann et al. 2006a) [11]. The aim of this study was to investigate the effect of pneumonia which may be acquired by the patients during their stay in the ICU. The data is publicly available in the format of *los.data* from the *etm* R package. Briefly, *los*.*data* includes 756 patients who are admitted to the ICU between February 2000 and July 2001. After having been admitted to the ICU, 124 (16.4*%*) patients acquired pneumonia (infection) in the hospital. Among those who got infected, 34 (27.4*%*) patients died. Overall, 191 patient died after ICU admission which is 25.3%. None of the patients were censored.

For the analysis, we first modify the data structure such that it corresponds to model A. Moreover, to analyze the cLOS under the “censored model” (model B), the information of the patients who died is censored at the time of their death. Table 1 shows an extract of the dataset under each model.

**Table 1:** Extract of the data showing the artificial censoring of the patients who died in the hospital at the time of their death, denoted by “cens”. It shows the patient identification number (“id”), transition state (“from” and “to”), time taken by the patient to move from state 0 to the current state “to” is given by “time”. State “1” defines when the patient is infected, state “2” defines when the patient is discharged alive and in model A, state “3” defines death of the patient at the hospital while the same patients are artificially censored in model B

Id	From	To	Time
Model A			
22	0	1	4
22	1	2	16
29	0	1	6
29	1	3	22
..	..	..	..
245	0	2	28
250	0	2	9
..	..	..	..
4	0	3	11
17	0	3	4
..	..	..	..
..	..	..	..
Model B			
22	0	1	4
22	1	2	16
29	0	1	6
29	1	cens	22
..	..	..	..
245	0	2	28
250	0	2	9
..	..	..	..
4	0	cens	11
17	0	cens	4
..	..	..	..
..	..	..	..

**Fig. 3:**
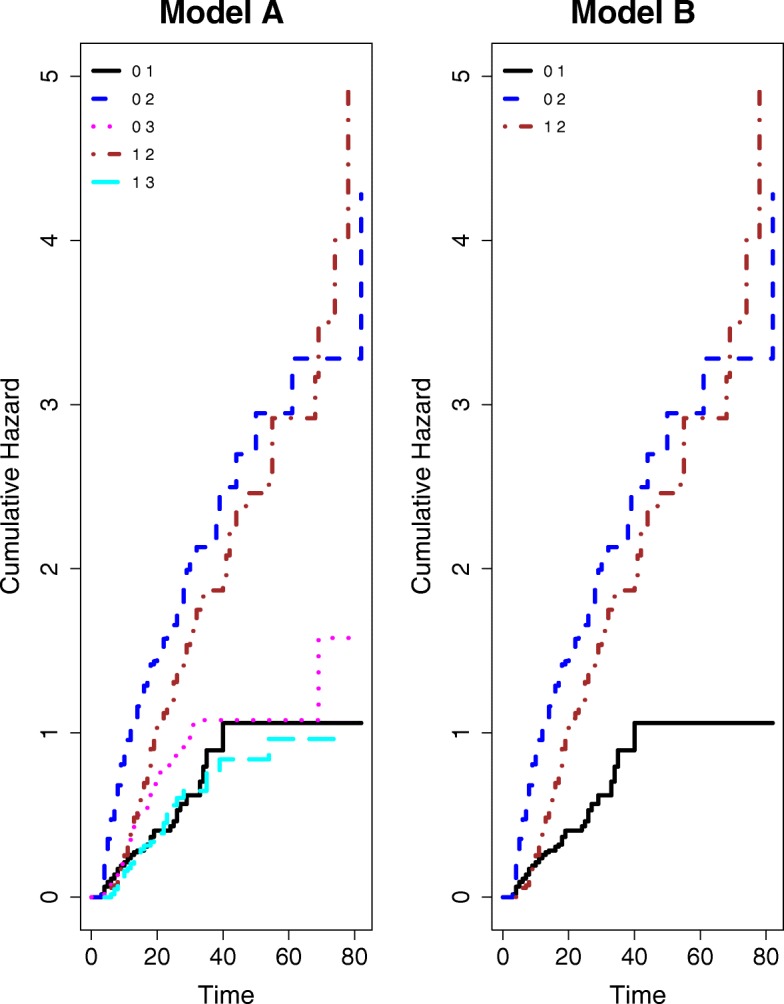
Estimated Cumulative hazards rates in the first 80 days for the multi-state models in Figs. 1 and 2. The slope of each line corresponds to the actual hazard rate, e.g a straight line would mean a constant hazard rate. The left figure shows the cumulative hazard functions for model A, when death is considered as competing event. The right figure corresponds to that of model B, when the patients are censored at the time of death

To obtain first insights into the data structure, we estimate the cause-specific cumulative hazards for model A (shown in Fig. 3). The graph indicates that the cumulative discharge hazards are not straight lines (which implies that they are not constant). Moreover, we observe that the discharge hazard is consistently reduced for patients with an HI. The cumulative hazards are estimated using the R-package *mvna*, developed by Allignol et al. (2008) [12] based on the Nelson-Aalen estimator. We also estimated the cumulative hazard rates for model B where the patients are censored at the time of their death (also shown in Fig. 3). We can clearly see that the censoring does not affect the other hazard rates. This means the discharge hazards as well as the infection hazard of model A and B are the same. Note that pneumonia appears to have no effect on the death hazard. However, this does not imply that pneumonia has no effect on mortality. The reason is that pneumonia reduces the discharge hazard as a consequence patient with pneumonia stay longer in the ICU. As a consequence, more patients with pneumonia are observed to die in the ICU than patients without pneumonia. The effects of HI on the death and discharge rates can be estimated with two cause-specific hazards model (for death and discharge). The indirect effect on mortality due to a decreased discharge hazard is commonly observed for hospital-acquired infections [13, 14].

### Data analysis on the effect of censoring on the estimated extra length of stay

In this section we estimate the cLOS of the SIR-3 data sample. We first use the non-parametric approach for time-inhomogeneous Markov models followed by the parametric approach assuming the hazard rates of the dataset are constant. In both approaches we estimate the cLOS by describing the data with model A and model B respectively. Then we calculated the magnitude of the bias occurring in model B.

Moreover, we distinguish the cLOS obtained from model A between patients being discharged alive and patients that die. This way we can investigate how many extra ICU days are attributable to patients being discharged alive. This quantity is also compared to the biased model where the cLOS attributable to discharged patients is estimated by treating patients that die as censored.

#### Non-parametric model

**Fig. 4:**
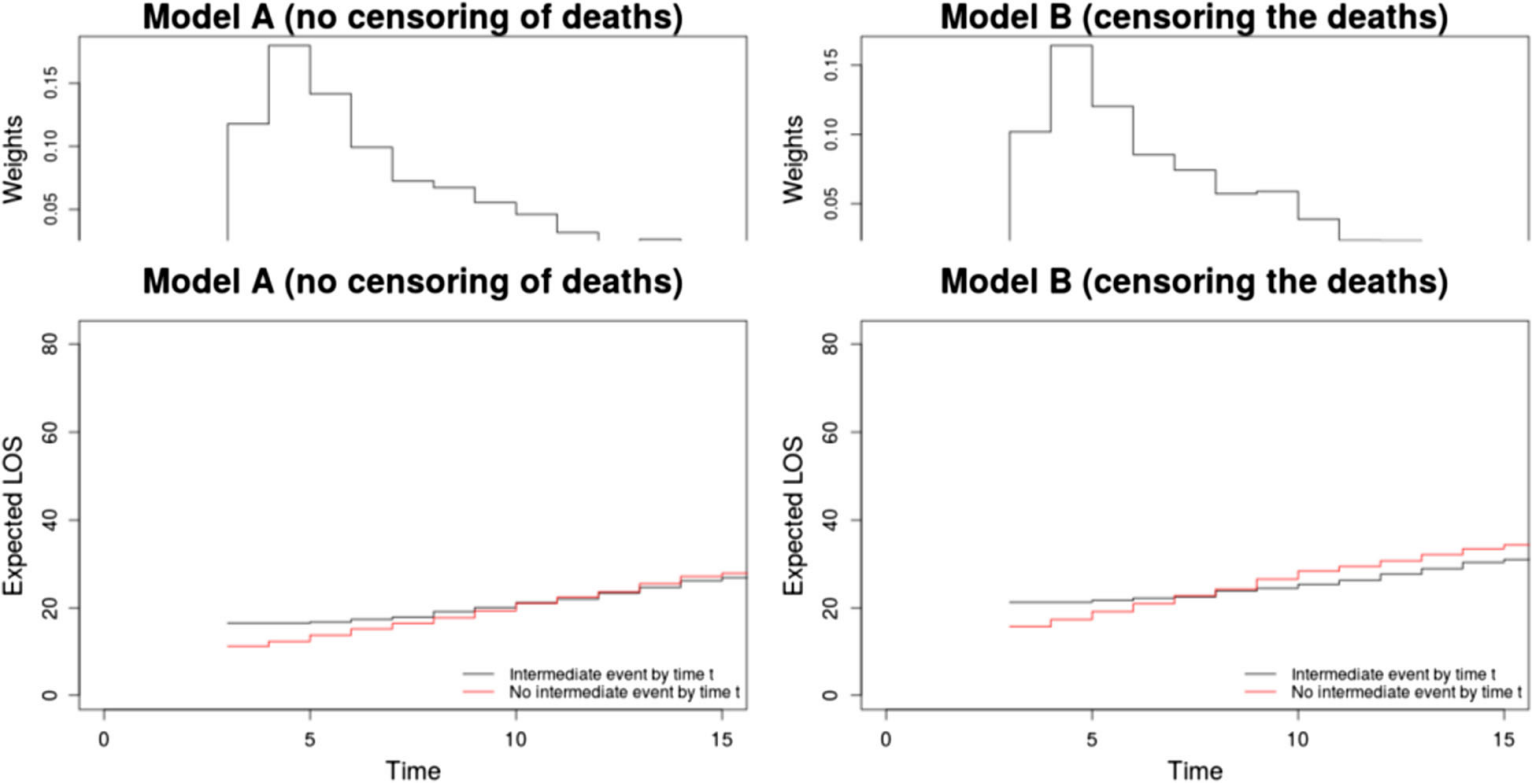
Weights and expected LOS for patients with and without an HI in the first 15 daysof los.data, which is a subset of the SIR-3 study. The left figure corresponds to model A (death cases are considered as competing event). The right figure corresponds to model B (death cases are censored). The estimated cLOS due to model A is 1.975 days and that for model B is 0.446 days

We estimate the difference in cLOS associated with HIs within the framework of model A (no censoring of death cases) and model B (censoring of patients at the time of their death) by using the R-package *etm*. The package is based on computing the Aalen-Johansen estimators assuming a time-inhomogeneous Markov model. For model A, the estimated cLOS due to HIs is greater for earlier days (see the lower graphs in Fig. 4). The average cLOS over all days is calculated by weighting the differences in length of stay on each day. This gives an estimated cLOS of 1.975 days. The corresponding weight distributions are also illustrated in Fig. 4. The average expected cLOS estimated after censoring the information of the patients at the time of their death is 0.446 days (model B). So the difference in the cLOS estimated from model B and model A using the R package *etm* is 1.529 days.

We note in Fig. 4 that for model B the estimate for the cLOS in hospitals with and without infection cross each other. This implies that the underlying assumption of a homogeneous Markov model, i.e, when the hazard rates are constant, may not be a viable assumption for the data set. As Allignol et al. noted, these curves should be parallel for the homogeneous Markov assumption to be plausible.

#### Parametric model with constant hazards

To compare and investigate the results from the *etm* package with the analytical expressions derived in “Results and discussion” section, we further estimate the cLOS by assuming that the hazard rates are constant.

We first estimate the constant hazard rates with Eq. (1). We obtain, $\hat {\alpha }_{01} = 0.019$, $\hat {\alpha }_{02} = 0.074$, $\hat {\alpha }_{03} = 0.024$, $\hat {\alpha }_{12} = 0.059$ and $\hat {\alpha }_{13} = 0.022$. Under a homogeneous Markov process, this data-situation is similar to indirect differential mortality. Plugging the estimates into the formulas in Eqs. (2) and (4), the cLOS from model A is 1.773. The cLOS due to HIs with censoring of the death cases is 2.699 (model B). Thus, censoring of the death cases is overestimating the cLOS by 0.926 days in the time-constant hazards set up. Unlike in the case of *etm*-estimation (time-inhomogenuous Markov model), in the time-constant hazards set up, model B is overestimating the cLOS with respect to model A.

Comparing the two estimation methods, we find that the cLOS under constant hazards is similar to the value obtained with the *etm*-package for model A (1.773 days and 1.975 days respectively). From model B, we obtain 2.699 days under the constant hazards assumption and 0.446 days with *etm*. Thus, the values obtained from model B clearly differ. While in the estimation with *etm*, model B is underestimating the cLOS with respect to model A, we observe the opposite under the constant hazards assumption.

This difference in behavior could be attributed to the consequence of the violation of the constant hazards assumption. As seen in Fig. 4, the cLOS of patients with and without HI cross for model B indicating a much stronger discrepancy to the assumption than for model A, where the curves rather touch than cross. These circumstances can further be understood when comparing the combined hazards with and without HI of model A and B with their time-constant counterparts shown in the Additional file 1: Figure S5.

For a more detail inspection, we estimate the cause-specific hazard rates non-parametrically with B-splines using the R-package *bshazard*. A detailed description of the method is given by Rebora et al. [15]. The estimated death and discharge hazards both with and without HI are shown in the Additional file 1: Figure S6. The plots show also the hazard rates obtained by using equation (1), where we assume that they are constant. Comparing the estimated hazard rates with their time-constant analogues we clearly see that the data does not correspond to a homogeneous Markov model. The discharge hazard before HI increases strongly in the first 10 days. After a peak at day 10 it strongly decreases again and remains on a moderate level from day 20 onward. The behavior of the death hazard before HI is similar but on a much lower level. Furthermore, it remains below the discharge hazard before HI at all time-points. In contrast, the discharge hazard after HI seems to be almost constant and is well approximated by $\hat {\alpha }_{12}$ from formula (1). The death hazard after HI continuously slightly decreases and always remains below the discharge hazard after HI.

While the constant hazards assumption is not plausible, the time-inhomogeneous Markov assumption is. Testing this assumption by including time of HI as covariate in a Cox regression model showed no effect on the death and discharge hazards after HI. The hazard ratios were 0.98 ([0.94 ; 1.01]) and 1.03 ([0.96 ; 1.09]) respectively.

#### Distinction between discharged (alive) and dead

Using the *clos* function in the *etm*-package, we obtain 1.998 days as the estimated cLOS attributable to patients who are discharged alive and −0.0234 days attributable to those who died in the ICU. The difference in the estimated cLOS for model B and that attributable to patients discharged alive under model A is therefore about 1.552 days. Thus, model B also underestimates cLOS attributable to patients discharged alive. It is further to be noted that under estimation with *etm* (model A) overall cLOS is similar to that of the cLOS estimated for discharged patients (model A). This is due to the circumstance that most of the patients are discharged alive.

Using the formula (3) for the constant hazards approach, we obtain 1.291 days for discharged patients and 0.4815 days for the deceased patients. In Table 2, we see that model B is overestimating the cLOS with respect to the cLOS (due to discharge alive) by 1.408 days. This means model B is clearly overestimating the estimate from cLOS using only the discharged patients assuming a time-constant hazards set up.

**Table 2:** Estimation of cLOS with respect to model A (no censoring of deaths) as well as cLOS (discharged) and cLOS (death) (based on model A but distinguishing between death and discharge). Moreover, cLOS with respect to model B (censoring of deaths). Additionally we calculate the bias between model A and model B and the bias between model B and cLOS based on model A for discharged patients only. The comparison is done for the estimation of cLOS by assuming constant hazard and by using the *etm* package (assuming time-dependent hazards)

	CLOS	CLOS	CLOS	CLOS^∗^	Bias	Bias
	(Model A)	discharged (Model A)	death (Model A)	(Model B)	(Model B - Model A)	(Model B - discharged only)
constant	1.773	1.291	0.482	2.699	0.926	1.408
hazard						
etm	1.975	1.998	-0.0234	0.446	-1.529	-1.552
package						

When comparing the time-inhomogeneous to the homogeneous (constant hazards) approach under model A we observe that the difference between overall cLOS and cLOS due to patients discharged alive is higher for the homogeneous approach. This is due to the circumstance that under constant hazards the effect of HI is averaged over the complete time-interval to estimate cLOS. Using the time-inhomogeneous approach by Allignol et al. cLOS is weighted according to the different lengths of stay. As most patients are discharged alive within the first few days, the weights are highest at these time-points (see Fig. 4). When using the homogeneous approach then the influence of the discharged patients on the estimate of cLOS is less strong.

The complete R code of the data analysis is provided in the Additional file 2.

## Conclusion

The major innovation of this study was the systematic evaluation of the bias due to censoring of death cases when studying cLOS in the hospital due to HIs. While Allignol et al. [7] provided an appropriate estimator, the existence of the bias due to censoring of death cases was neither mentioned nor discussed by the authors.

We first evaluated the bias in a mathematically closed form assuming a setting with constant hazards. A similar approach in a simpler setting without competing outcomes has been used by Joly et al. [8]. Our analytical evaluation has the advantage that we are able to discuss challenging effects regarding direct and indirect differential mortality. Moreover, it allows us to make statements about the magnitude and direction of the bias.

The real data application also showed that effects regarding direct and indirect differential mortality do exist and that the bias influences the estimates of cLOS. In model A, the cLOS estimation via the time-homogeneous model gave similar estimates as the one which allows time-inhomogeneity, whereas it was different for model B where we treated patients that die as censored observations. Although a difference in estimation of cLOS has been observed due to censoring of death cases both by using the “time-dependent hazard” (via *etm* package) and the “time-constant hazard” assumptions, the bias shown in the two set ups goes in different direction. Thus, our closed formula has limitations if the assumptions are not fulfilled. Therefore, a time-dependent hazards model should be considered for future research. However, before dealing with a complicated time-inhomogeneous model one must understand the behavior of the bias for the simpler constant hazards model. Understanding the bias in a simple setting was the aim of this paper. To point out the presence of bias in a real world situation we have used the publicly available SIR-data. Even thought the constant hazards assumption is not possible for this data set we could demonstrate the existence of the bias.

A further limitation of our study was not considering confounding factors as the length of stay of the patients may depend on the underlying morbidity of the patient. We emphasize that the bias due to censoring the death cases is a type of survival bias and systematically different from confounding. Based on our findings, we can conclude that censoring the deaths should be avoided. Moreover, the formula we derived can be used to describe the bias for settings with constant hazards.

## Additional files

Additional file 1 The document contains **Figures S5** and **S6** mentioned in section ’Results and discussion’ as well as the detailed mathematical derivation of the bias formula presented in section ’Methods’. (PDF 192 kb)

Additional file 2 The document is the complete R script used in section 4 for the data analysis. (R 6 kb)

## Abbreviations

ICU: Intensive care unit
HI: Hospital-acquired infection
cLOS: change in length of hospital/ICU stay
