# Supplementary material for: Bias due to censoring of deaths when calculating extra length of stay for patients acquiring a hospital infection
Source: BMC Med Res Methodol. 2018 May 30;18:49. doi: 10.1186/s12874-018-0500-3 (PMC5975458; doi:10.1186/s12874-018-0500-3)
Supplement: Supplementary file 1 — The document contains Figures S5 and S6 mentioned in section ’Results and discussion’ as well as the detailed mathematical derivation of the bias formula presented in section ’Methods’. (PDF 192 kb) [file 12874_2018_500_MOESM1_ESM.pdf]

RESEARCH

Bias due to Censoring of Deaths when Calculating Extra Length of Stay for Patients Acquiring a Hospital Infection - Supplementary Material

Shahina Rahman, Maja von Cube, Martin Schumacher and Martin Wolkewitz

Supplementary Material

Figures 5 and 6

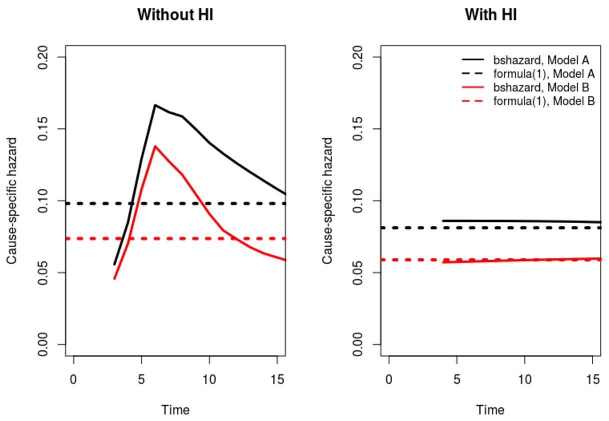

Figure 1 The left panel shows the combined hazard rates for model A (back line) and model B (red line) before the occurrence of an HI. The solid lines are the combined hazard rates corresponding to the data example los.data estimated with etm, the dashed lines are their time constant analogues obtained with formula (1). The right panel shows correspondingly the combined hazard rates after the occurrence of HI. For model A, the combined hazards are the sum of the death and discharge hazards with and without HI. For model B, as the event of death is ignored, this is only the discharge hazard with and without HI respectively.

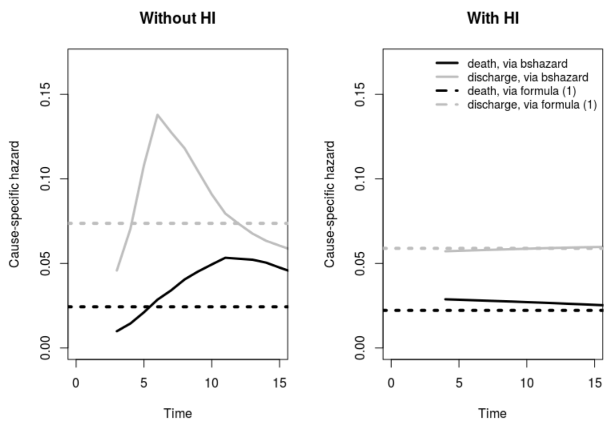

Figure 2 The left panel displays the cause-specific hazard rates of death (black line) and discharge (grey line) before the occurrence of HI. The solid lines are the hazard rates corresponding to the data example los.data estimated with etm, the dashed lines are their time constant analogues obtained with formula (1). The right panel shows correspondingly the cause-specific hazard rates of death and discharge after the occurrence of HI.

Proof of the Bias calculation

From equation (2) and (4) of the main article

$$\mathrm{CLOS}_{\mathrm{true}} = \Big[\frac{\alpha_{02}+\alpha_{03}}{\alpha_{1\cdot}} - 1\Big]\frac{1}{\alpha_{0\cdot}}$$

where, $\alpha_{0\cdot} = \alpha_{01}+\alpha_{02}+\alpha_{03}$ and $\alpha_{1\cdot} = \alpha_{12}+\alpha_{13}$. Then the difference of length of stay for model B (Figure 2) when the incidence of deaths are censored is,

$$\mathrm{CLOS}^{*} = \Big[\frac{\alpha_{02}}{\alpha^{*}_{1\cdot}} - 1\Big]\frac{1}{\alpha^{*}_{0\cdot}}$$

where $\alpha^{*}_{0\cdot} = \alpha_{01}+\alpha_{02}$ and $\alpha^{*}_{1\cdot} = \alpha_{12}$. So we calculate,

$$\mathrm{CLOS}^{*} - \mathrm{CLOS}_{\mathrm{true}} = \Big[\frac{\alpha_{02}}{\alpha^{*}_{1\cdot}.\alpha^{*}_{0\cdot}} - \frac{\alpha_{02}}{\alpha_{1\cdot}.\alpha_{0\cdot}}\Big] - \frac{\alpha_{03}}{\alpha_{1\cdot}.\alpha_{0\cdot}} + \Big[\frac{1}{\alpha_{0\cdot}} - \frac{1}{\alpha^{*}_{0\cdot}}\Big].$$

We replace

$$\frac{1}{\alpha_{0\cdot}} - \frac{1}{\alpha^{*}_{0\cdot}} = -\frac{\alpha_{03}}{\alpha_{0\cdot}.\alpha^{*}_{0\cdot}}. \qquad (1)$$

Thereafter,

$$\begin{aligned}
\mathrm{CLOS}^* - \mathrm{CLOS}_{\mathrm{true}} &= \alpha_{02}\Big[\frac{1}{\alpha^*_{1\cdot}\alpha^*_{0\cdot}} - \frac{1}{\alpha_{1\cdot}\alpha_{0\cdot}}\Big] - \alpha_{03}\Big[\frac{1}{\alpha_{1\cdot}\alpha_{0\cdot}} + \frac{1}{\alpha_{0\cdot}\alpha^*_{0\cdot}}\Big],\\
&= \frac{\alpha_{02}}{\alpha_{12}\alpha_{0\cdot}}\Big[\frac{\alpha_{0\cdot}}{\alpha^*_{0\cdot}} - \frac{\alpha_{12}}{\alpha_{1\cdot}}\Big] - \frac{\alpha_{03}}{\alpha_{12}\alpha_{0\cdot}}\Big[\frac{\alpha_{12}}{\alpha_{1\cdot}} + \frac{\alpha_{12}}{\alpha^*_{0\cdot}}\Big],\\
&= \frac{\alpha_{02}\alpha_{0\cdot}}{\alpha_{12}\alpha_{0\cdot}\alpha^*_{0\cdot}} - \frac{\alpha_{03}\alpha_{12}}{\alpha_{12}\alpha_{0\cdot}\alpha^*_{0\cdot}} - \frac{\alpha_{03}\alpha_{12}}{\alpha_{12}\alpha_{0\cdot}\alpha^*_{0\cdot}} - \frac{\alpha_{03}\alpha_{12}}{\alpha_{12}\alpha_{0\cdot}\alpha_{1\cdot}},\\
&= \frac{\alpha_{02}\alpha_{0\cdot} - \alpha_{03}\alpha_{12}}{\alpha_{12}\alpha_{0\cdot}\alpha^*_{0\cdot}} - \frac{\alpha_{12}(\alpha_{02}+\alpha_{03})}{\alpha_{12}\alpha_{0\cdot}\alpha_{1\cdot}},\\
&= \frac{\alpha_{02}(\alpha_{01}+\alpha_{02}+\alpha_{03}) - \alpha_{03}\alpha_{12}}{\alpha_{12}\alpha_{0\cdot}\alpha^*_{0\cdot}} - \frac{\alpha_{02}+\alpha_{03}}{\alpha_{0\cdot}\alpha_{1\cdot}},\\
&= \frac{\alpha_{03}(\alpha_{02}-\alpha_{12})}{\alpha_{12}\alpha_{0\cdot}\alpha^*_{0\cdot}} + \frac{\alpha_{02}(\alpha_{01}+\alpha_{02})}{\alpha_{12}\alpha_{0\cdot}\alpha^*_{0\cdot}} - \frac{\alpha_{02}+\alpha_{03}}{\alpha_{0\cdot}\alpha_{1\cdot}},
\end{aligned}$$

Since $\alpha^*_{0\cdot} = \alpha_{01} + \alpha_{02}$, we have the bias formula in equation (**??**)

$$\begin{aligned}
\mathrm{CLOS}^* - \mathrm{CLOS}_{\mathrm{true}} &= \frac{\alpha_{03}(\alpha_{02}-\alpha_{12})}{\alpha_{12}\alpha_{0\cdot}\alpha^*_{0\cdot}} + \frac{\alpha_{02}}{\alpha_{12}\alpha_{0\cdot}} - \frac{(\alpha_{02}+\alpha_{03})}{\alpha_{0\cdot}\alpha_{1\cdot}}\\
&= \frac{\alpha_{03}(\alpha_{02}-\alpha_{12})}{\alpha_{12}\alpha_{0\cdot}\alpha^*_{0\cdot}} + \frac{\alpha_{02\alpha_{1\cdot}}}{\alpha_{12}\alpha_{0\cdot}\alpha_{1\cdot}} - \frac{\alpha_{12}(\alpha_{02}+\alpha_{03})}{\alpha_{12}\alpha_{0\cdot}\alpha_{1\cdot}}\\
&= \frac{\alpha_{03}(\alpha_{02}-\alpha_{12})}{\alpha_{12}\alpha_{0\cdot}\alpha^*_{0\cdot}} + \frac{(\alpha_{02}\alpha_{13}-\alpha_{03}\alpha_{12})}{\alpha_{12}\alpha_{0\cdot}\alpha_{1\cdot}}.
\end{aligned}$$

No Differential Mortality

Under $\Delta_1 = \alpha_{13} - \alpha_{03} = 0$ and $\Delta_2 = \alpha_{02} - \alpha_{12} = 0$, $\mathrm{CLOS}_{\mathrm{true}} = \mathrm{CLOS}^* = 0$. Hence the Bias is 0.

Direct Differential Mortality

When $\Delta_2 = 0$, i.e, substituting $\alpha_{02} = \alpha_{12}$, we get the direct bias in equation (**??**),

$$\mathrm{CLOS}^* - \mathrm{CLOS}_{\mathrm{true}} = \frac{\alpha_{02}(\alpha_{13}-\alpha_{03})}{\alpha_{12}\alpha_{0\cdot}\alpha_{1\cdot}} = (\alpha_{13}-\alpha_{03})\cdot\frac{1}{\alpha_{0\cdot}}\cdot\frac{1}{\alpha_{1\cdot}}.$$

Indirect Differential Mortality

We start with adjusting the numerator and denominators to obtain same denominator,

$$\mathrm{CLOS}^* - \mathrm{CLOS}_{\mathrm{true}} = \frac{\alpha_{1\cdot}\cdot\alpha_{03}(\alpha_{02}-\alpha_{12})}{\alpha_{12}\alpha_{0\cdot}\alpha^*_{0\cdot}\alpha_{1\cdot}} + \frac{\alpha^*_{0\cdot}\cdot(\alpha_{02}\alpha_{13}-\alpha_{03}\alpha_{12})}{\alpha_{12}\alpha_{0\cdot}\alpha^*_{0\cdot}\alpha_{1\cdot}}.$$

Under indirect differential mortality, we have, $\Delta_1 = 0$, i.e. when $\alpha_{03} = \alpha_{13}$, we get the equation (6) of the main article,

$$\begin{aligned}
\mathrm{CLOS}^* - \mathrm{CLOS}_{\mathrm{true}} &= \frac{\alpha_{1\cdot}\alpha_{03}(\alpha_{02} - \alpha_{12})}{\alpha_{12}\alpha_{0\cdot}\alpha^*_{0\cdot}\alpha_{1\cdot}} + \frac{\alpha^*_{0\cdot}\alpha_{03}(\alpha_{02} - \alpha_{12})}{\alpha_{12}\alpha_{0\cdot}\alpha^*_{0\cdot}\alpha_{1\cdot}}, \\
&= \frac{(\alpha_{02} - \alpha_{12})\alpha_{03}(\alpha_{12} + \overline{\alpha_{13} + \alpha_{01} + \alpha_{02}})}{\alpha_{12}\alpha_{0\cdot}\alpha^*_{0\cdot}\alpha_{1\cdot}}, \\
&= \frac{(\alpha_{02} - \alpha_{12})\alpha_{03}(\alpha_{12} + \alpha_{0\cdot})}{\alpha_{12}\alpha_{0\cdot}\alpha^*_{0\cdot}\alpha_{1\cdot}}. \\
&= (\alpha_{02} - \alpha_{12}) \cdot \frac{1}{\alpha_{0\cdot}} \cdot \frac{1}{\alpha_{1\cdot}} \cdot \frac{\alpha_{03}(\alpha_{0\cdot} + \alpha_{12})}{\alpha_{12}(\alpha_{01} + \alpha_{02})}.
\end{aligned}$$
